# Substance Use Among American Indian Youths on Reservations Compared With a National Sample of US Adolescents

**DOI:** 10.1001/jamanetworkopen.2018.0382

**Published:** 2018-05-31

**Authors:** Randall C. Swaim, Linda R. Stanley

**Affiliations:** 1 Colorado State University, Fort Collins

## Abstract

**Importance:**

American Indian adolescents attending schools on or near reservations are historically at high risk for substance use.

**Objective:**

To compare rates of substance use among reservation-based American Indian adolescents vs rates among national US youths.

**Design, Setting, and Participants:**

Population-based survey study of 8th-, 10th-, and 12th-grade students attending participating schools on or near reservations, stratified by region, during the 2016-2017 school year. Substance use rates were compared with those of a national sample of comparably aged students from the Monitoring the Future study.

**Main Outcomes and Measures:**

Lifetime and last-30-day self-reported use of alcohol, marijuana, and other drugs, using relative risk (RR) ratios with 95% confidence intervals to compare American Indian student rates with Monitoring the Future student rates.

**Results:**

Participants included 570 students in eighth grade (49.6% girls; mean age, 13.5 years), 582 in 10th grade (50.0% girls; mean age, 15.4 years), and 508 in 12th grade (53.5% girls; mean age, 17.4 years). American Indian students reported substantially higher lifetime and last-30-day substance use rates compared with the Monitoring the Future students, with greatest disparity at eighth grade: last-30-day substance use RRs for grade 8 were 2.1 (95% CI, 1.4-3.0) for alcohol, 4.2 (95% CI, 3.1-5.8) for marijuana, and 2.4 (95% CI, 1.7-3.3) for other illicit drugs. Compared with 2009 to 2012 data, the RRs between American Indian and Monitoring the Future students for lifetime alcohol and marijuana use did not change substantially from the 2016-2017 school year (alcohol: RR, 1.5 [95% CI, 1.4-1.6] vs RR, 1.3 [95% CI, 1.2-1.4], respectively; marijuana: RR, 2.0 [95% CI, 1.8-2.1] vs RR, 2.1 [95% CI, 1.9-2.3], respectively), but increased substantially for other drugs (RR, 1.8 [95% CI, 1.7-1.9] vs RR, 3.0 [95% CI, 2.9-3.2], respectively).

**Conclusions and Relevance:**

Reservation-based American Indian students are at high risk for substance use compared with US youths in general, making prevention efforts critical. Cultural and value-based characteristics unique to American Indian populations may provide beneficial targets for prevention, but there is limited evidence on how cultural factors work to prevent risky behaviors. Without increased attention to these disparities, the costs to American Indian youths and their communities will remain high.

## Key Points

**Question:**

How do substance use rates among American Indian youths compare with rates among national US adolescents?

**Findings:**

Lifetime and last-30-day substance use rates and relative risk were significantly higher for nearly all substances among American Indian youths.

**Meaning:**

Early prevention and culturally sensitive interventions are needed for this population in addition to careful screening by medical staff for signs of early initiation and substance abuse and dependence.

## Teaser

This population-based survey study compares rates of use of alcohol, marijuana, cigarettes, and illicit drugs among eighth-grade and high school reservation-based American Indian adolescents with rates among a national sample of US adolescents in 2016-2017.

## Introduction

We have tracked rates of substance use among American Indian adolescents attending schools on or near reservations since 1974, and American Indian adolescents have consistently reported the highest levels of substance use compared with other US racial/ethnic groups.^1,2,3,4^ Other studies of mostly nonreservation American Indian youths report similar results.^5,6,7^ When compared with a national sample of students from Monitoring the Future (MTF), our study of data from 2009 to 2012 indicated that American Indian students reported lifetime rates of marijuana 3.4 and 1.6 times higher for 8th- and 12th-grade students, respectively.^8^ Differences for current marijuana use for these grades were larger, at 4.8 to 1.6 times higher, respectively. An important consideration for American Indian youths is that, in addition to high rates of substance use, risk for substance use begins early.^9,10,11^ As previously reported, we compared initiation rates of alcohol, marijuana, and inhalants between a population-based sample of reservation-based American Indian youths with white youths attending the same schools, and odds ratios for initiation comparing these 2 groups were 2.3 (95% CI, 1.5-3.4) for intoxication, 10.5 (95% CI, 6.0-18.5) for marijuana, and 1.8 (95% CI, 1.3-2.4) for inhalants, with American Indian youths more likely to initiate early use.^12^

The harm associated with high rates of use and early initiation for American Indian youths include increasing rates of use in early and later adulthood, higher risk of developing a substance use disorder, and more alcohol-related problems, including alcohol-attributable death.^7,13,14^ Furthermore, American Indian and Alaska Native youths are more likely to need treatment for a substance use problem than all other US racial/ethnic groups.^15^ These findings underscore the need for continuing surveillance of this at-risk group, particularly given changing trends in perceived harmfulness of illicit substances as new statutes alter access to medical and recreational use of cannabis.^16,17^

Our previous surveys corresponded closely to items from the MTF study, an ongoing population-based study of US youths. In coordination with the National Institute on Drug Abuse Epidemiology Research Branch and MTF staff, we revised our survey (Our Youth, Our Future [OYOF]) to use identical items from MTF, making direct comparisons possible. This allows for ongoing comparisons of substance use rates between population-based samples of reservation-based American Indian students and national US students.

## Methods

### Sample and Recruitment

The data are part of an ongoing epidemiologic study of substance use by American Indian youths living on or near reservations, where students in grades 7 through 12 in sampled schools complete online surveys of substance use. The sampling frame was built from 3 primary sources: the National Center for Education Statistics Common Core of Data, the National Center for Education Statistics Private School Universe Survey, and the Bureau of Indian Education National Directory. We followed the American Association for Public Opinion Research (AAPOR) reporting guideline. The final sampling frame contained 353 schools that included students in seventh grade or higher, located on or within 25 miles of a reservation or tribal lands, with at least 20% of students enrolled being American Indian. Schools outside the continental United States were excluded, as were Oklahoma tribal statistical areas. Schools were stratified into 7 regions (Northeast, Northwest, Southeast, Southwest, Northern Plains, Southern Plains, and Upper Great Lakes) based on cultural and other similarities among American Indian groups.^18^

Each year schools within each region are randomly drawn from the sampling frame to reflect the regional distribution of American Indian persons residing in each stratum based on the US 2010 Census data. Because of the small number of schools in the Northeast, Southern Plains, and Southeast, all schools and/or school districts meeting the requirements of the sample are invited to participate. For schools not including high school grades (eg, middle schools), the high school most likely to be attended by the American Indian middle school students is determined and added to the drawn sample. Participating schools were reasonably representative of sampled schools in several measurable demographic characteristics. Approximately 28% of sampled schools and 21% of surveyed schools were Bureau of Indian Education schools, while 67% of sampled schools and 79% of participating schools were public. Middle schools composed 27% of sampled schools and 21% of surveyed schools; K-12 schools were 11% of sampled schools and 10% of participating schools; and K-8 (or similar) schools were 27% of sampled schools and 21% of surveyed schools. Schools with greater than 80% American Indian students composed 50% of sampled schools and 41% of participating schools, and schools on reservations were 65% of the sample vs 55% of participating schools.

For each participating school, the appropriate tribal and school board approvals were obtained. Each school received a comprehensive report of their survey findings and compensation for resources used to complete the survey process, ranging from $750 to $5000 depending on enrollment, with median payment being $1500.

All survey responses were collected anonymously, and all procedures were approved by the Colorado State University institutional review board. The board approved waiver of signed parental consent; however, assent was obtained from both parents and students.

### Participants

This study used survey data from participating schools during fall 2016 to spring 2017 semesters of the 2016-2017 academic year. Specific identities of tribes and reservations were kept confidential. Across participating schools, the number of completed surveys (n = 6065) represented 87.7% of student enrollment in those schools. To make direct comparisons with MTF, only students in 8th, 10th, and 12th grade who self-identified as American Indian were included in the analysis.

Sample sizes for MTF ranged from 8450 to 16 900 for eighth graders, 7350 to 15 900 for 10th graders, and 3933 to 11 800 for 12th graders, depending on the substance. The number of cases varied by substance because multiple questionnaire forms are randomly distributed at each grade level, and not all questions are asked on all forms to reduce response burden.^19^

### Procedure

Approximately 3 weeks before the scheduled survey, letters were sent to parents of enrolled students in grades 7 and higher describing the survey and providing instructions for opting their child out of the survey. This information was also posted on other local media sites, where parents were likely to see it. Less than 1% of students refused to take the survey or were opted out by their parents.

The OYOF survey was administered online to students using Qualtrics software. School staff read directions prior to survey administration indicating that students could decline to participate or leave blank any questions they did not wish to answer, and these instructions were repeated in the online survey. Staff were instructed to remain in an area of the room where they could not observe students’ responses.

### Measures

The OYOF survey contains a verbatim subset of the most recent substance use questions asked in the MTF survey. For each substance, the OYOF survey asks about lifetime and last-30-day use in addition to measures of demographic characteristics. Substance use measures for each question were coded as 1 for any use and 0 for no use. In addition to single substance use measures, a composite measure of illicit drug use was calculated, using the MTF definition, as any use of lysergic acid diethylamide (LSD), other hallucinogens, crack, cocaine other than crack, heroin, any use of narcotics other than heroin (for 12th grade only), amphetamines, sedatives (barbiturates), or tranquilizers not under a physician’s orders.

### Statistical Analysis

Demographic characteristics and outcome data are reported using descriptive statistics, proportions with 95% confidence intervals, and relative risk (RR) ratios with 95% confidence intervals. For each outcome measure at each grade, lifetime and last-30-day use prevalence rates and their 95% confidence intervals were computed, excluding missing data, using Stata version 15 (StataCorp) survey commands, with stratification by region and with school as the primary sampling unit. Missingness varied between 0.4% and 2.6% for individual substance measures except hallucinogens other than LSD, for which missing rates varied from 4.8% for grade 10 to 14.4% for grade 8. This was because 1 tribal group requested that this question not be asked. Observations were weighted to correct for overrepresentation or underrepresentation by region, with weights based on the US 2010 Census reservation and off-reservation trust land state populations for ages 10 to 19 years. Weights varied from 0.39 (Northeast) to 2.25 (Southeast), with the remaining weights between 0.70 and 1.30. Comparable MTF substance use rates were obtained from MTF’s National Survey Results for Drug Use 1975 to 2016.^19^

The RRs comparing American Indian and MTF students’ lifetime and last-30-day use prevalence rates and their 95% confidence intervals with a test of significant difference from 1 (*P* < .05, 2-sided) were calculated using MedCalc statistical software version 16.4.3 (MedCalc Software). To account for nesting of students within schools, effective sample sizes for each substance within a sample were computed as actual sample size divided by design effect for that substance.^20^ We compared lifetime and last-30-day use RRs from our 2009 to 2012 data^6^ with the current RRs averaged across the 3 grades for alcohol, marijuana, and other drugs. A composite alcohol RR was calculated as the mean alcohol, intoxication, and binge drinking RR (for last 30 days), while the other-drug RR was the mean RR for all other drugs except marijuana.

## Results

Thirty-one schools participated in the OYOF survey with the following regional distribution of students: Northeast, 6.1%; Northwest, 9.7%; Northern Plains, 20.5%; Southeast, 9.9%; Southwest, 43.3%; and Upper Great Lakes, 10.6%. A total of 87.7% of enrolled students were surveyed. Participants included 570 students in eighth grade (49.6% girls; mean [SD] age, 13.5 [0.71] years), 582 in 10th grade (50.0% girls; mean [SD] age, 15.4 [0.67] years), and 508 in 12th grade (53.5% girls; mean [SD] age, 17.4 [0.70] years).

Table 1 and Table 2 present lifetime and last-30-day substance use prevalence rates for 8th-, 10th-, and 12th-grade students in the American Indian and MTF samples. In addition to point estimates and confidence intervals for American Indian students, the RR ratios are also presented, along with 95% confidence intervals and a test of significant difference from 1 (*P* < .05, 2-sided).

**Table 1.** Lifetime Prevalence of Alcohol and Drug Use Comparing Reservation-Based American Indian Students (2016-2017) With MTF Students (2016)

Type of Substance Use	Grade 8			Grade 10			Grade 12		
	American Indian, % (95% CI)	MTF, %^a^	RR (95% CI)	American Indian, % (95% CI)	MTF, %^a^	RR (95% CI)	American Indian, % (95% CI)	MTF, %^a^	RR (95% CI)
Alcohol	39.7 (31.4-48.6)	22.8	1.7 (1.4-2.2)^b^	52.9 (46.5-59.2)	43.4	1.2 (1.1-1.4)^b^	72.5 (66.0-78.1)	61.2	1.2 (1.1-1.3)^b^
Been drunk	22.9 (17.3-29.7)	8.6	2.7 (2.0-3.5)^b^	39.2 (31.6-47.4)	26.0	1.5 (1.2-1.9)^b^	56.5 (49.2-63.5)	46.3	1.2 (1.0-1.4)^b^
Marijuana	43.7 (35.1-52.7)	12.8	3.4 (2.8-4.2)^b^	55.6 (46.7-64.2)	29.7	1.9 (1.6-2.2)^b^	66.4 (57.3-74.5)	44.5	1.5 (1.3-1.7)^b^
Any illicit drug, not marijuana^c^	16.2 (12.7-20.5)	8.9	1.8 (1.5-2.3)^b^	19.1 (12.8-27.6)	14.0	1.4 (0.9-2.0)	24.4 (18.8-31.0)	20.7	1.2 (0.9-1.5)
Inhalants	13.2 (9.7-17.7)	7.7	1.7 (1.3-2.3)^b^	10.7 (8.6-13.1)	6.6	1.6 (1.3-2.1)^b^	10.8 (8.0-14.3)	5.0	2.2 (1.3-3.5)^b^
Tranquilizers	3.6 (2.2-5.7)	3.0	1.2 (0.8-2.0)	6.2 (3.6-10.7)	6.1	1.0 (0.6-1.7)	5.0 (3.5-7.1)	7.6	0.7 (0.5-0.9)^b^
Narcotics other than heroin	3.0 (2.0-4.5)	NA	NA	8.1 (5.2-12.3)	NA	NA	10.9 (7.6-15.4)	7.8	1.4 (0.9-2.0)
Amphetamines	4.0 (2.3-6.8)	5.7	0.7 (0.5-1.2)	5.8 (3.5-9.4)	8.8	0.7 (0.4-1.1)	10.0 (5.9-16.4)	10.0	1.0 (0.7-1.5)
Cocaine	4.3 (2.8-6.5)	1.1	3.9 (2.3-6.5)^b^	6.4 (3.6-11.0)	1.9	3.4 (1.8-6.0)^b^	11.8 (8.3-16.4)	3.3	3.6 (2.3-5.4)^b^
Crack	3.0 (1.7-5.2)	0.9	3.3 (1.8-5.7)^b^	3.8 (2.3-6.2)	0.8	4.8 (2.8-8.2)^b^	5.6 (3.4-9.3)	1.4	4.0 (2.2-6.9)^b^
LSD	3.9 (2.6-5.9)	1.2	3.3(2.1-5.1)^b^	6.1 (3.6-10.3)	3.2	1.9 (1.2-3.3)^b^	9.8 (6.3-14.9)	4.9	2.0 (1.2-3.2)^b^
Hallucinogens other than LSD	8.1 (5.6-11.6)	1.9	4.3 (2.8-6.4)^b^	9.9 (7.3-13.4)	3.1	3.2 (2.3-4.4)^b^	12.3 (8.6-17.4)	4.7	2.8 (1.9-4.1)^b^
Heroin	2.8 (1.8-4.3)	0.5	5.6 (3.3-9.8)^b^	2.4 (1.4-3.9)	0.6	4.0 (2.3-7.4)^b^	3.2 (2.2-4.6)	0.7	4.6 (2.9-7.3)^b^
Crystal meth^d^	2.6 (1.4-4.8)	0.6	4.3 (2.2-7.9)^b^	5.3 (4.0-7.0)	0.7	7.6 (5.1-10.8)^b^	8.0 (4.2-14.7)	1.4	5.7 (3.0-12.6)^b^
Cigarettes	29.7 (22.4-38.1)	9.8	3.0 (2.3-4.0)^b^	42.0 (36.2-47.9)	17.5	2.4 (2.0-2.8)^b^	49.7 (44.2-55.1)	28.3	1.8 (1.6-2.01)^b^

Abbreviations: LSD, lysergic acid diethylamide; MTF, Monitoring the Future; NA, not available; RR, relative risk.

^a^ Confidence intervals for MTF data can be found in Miech et al,^19^ Tables 4-1a through d.

^b^ *P* < .05.

^c^ Use of any illicit drug includes any use of LSD, other hallucinogens, crack, cocaine other than crack, heroin, any use of narcotics other than heroin (grade 12 only), amphetamines, or tranquilizers not under a physician’s orders.

^d^ Numbers for MTF grades 8 and 10 are for methamphetamine (including crystal meth).

**Table 2.** Last-30-Day Prevalence of Alcohol and Drug Use Comparing Reservation-Based American Indian Students (2016-2017) With MTF Students (2016)

Type of Substance Use	Grade 8			Grade 10			Grade 12		
	American Indian, % (95% CI)	MTF, %^a^	RR (95% CI)	American Indian, % (95% CI)	MTF, %^a^	RR (95% CI)	American Indian, % (95% CI)	MTF, %^a^	RR (95% CI)
Alcohol	15.8 (10.7-22.7)	7.3	2.1 (1.4-3.0)^b^	24.1 (20.0-28.7)	19.9	1.2 (1.0-1.5)	30.7 (25.1-36.9)	33.2	0.9 (0.8-1.1)
Been drunk	9.6 (5.8-15.4)	1.8	5.3 (3.3-8.9)^b^	16.5 (12.9-20.8)	9.0	1.8 (1.4-2.4)^b^	23.2 (17.7-29.8)	20.4	1.1 (0.8-1.5)
Binge drinking	11.8 (6.4-20.6)	3.4	3.5 (2.0-6.0)^b^	16.6 (13.6-20.0)	9.7	1.7 (1.4-2.1)^b^	22.8 (18.3-28.1)	15.5	1.5 (1.2-1.9)^b^
Marijuana	22.5 (16.1-30.5)	5.4	4.2 (3.1-5.8)^b^	35.1 (28.2-42.8)	14.0	2.5 (2.0-3.1)^b^	39.3 (32.1- 46.9)	22.5	1.7 (1.4-2.2)^b^
Any illicit drug, not marijuana^c^	6.4 (4.6-8.9)	2.7	2.4 (1.7-3.3)^b^	6.7 (3.8-11.7)	4.4	1.5 (0.9-2.7)	9.7 (7.0-13.3)	6.9	1.4 (0.9-2.0)
Inhalants	4.9 (3.4-7.2)	1.8	2.7 (1.8-4.1)^b^	2.2 (1.2-3.9)	1.0	2.2 (1.2-4.3)^b^	2.1 (1.2-3.6)	0.8	2.6 (1.2-6.1)^b^
Tranquilizers	1.4 (0.7-2.9)	0.8	1.8 (0.9-3.5)	1.6 (0.9-3.0)	1.5	1.1 (0.5-1.9)	2.7 (1.8-4.2)	1.9	1.4 (0.9-2.3)
Narcotics other than heroin	1.3 (0.7-2.3)	NA	NA	2.8 (1.6-4.9)	NA	NA	4.9 (2.8-8.2)	1.7	2.9 (1.6-5.2)^b^
Amphetamines	1.6 (0.8-3.4)	1.7	0.9 (0.5-1.8)	2.5 (1.5-4.1)	2.7	0.9 (0.5-1.6)	4.5 (2.8-7.3)	3.0	1.5 (0.8-2.6)
Cocaine	1.2 (0.6-2.5)	0.3	4.0 (1.9-9.4)^b^	2.4 (1.3-4.4)	0.3	8.0 (3.7-17.6)^b^	4.1 (2.8-6.1)	0.6	6.8 (3.5-13.1)^b^
Crack	0.8 (0.3-2.1)	0.2	4.0 (1.5-10.1)^b^	1.3 (5.9-2.9)	0.2	6.5 (2.5-15.0)^b^	1.7 (0.6-4.8)	0.5	3.4 (1.6-12.3)^b^
LSD	1.5 (0.8-2.8)	0.4	3.8 (2.3-9.1)^b^	1.5 (0.6-4.2)	0.7	2.1 (0.8-6.4)	2.5 (1.4-4.6)	1.0	2.5 (1.2-5.1)^b^
Hallucinogens other than LSD	2.2 (1.3-3.8)	0.3	7.3 (4.1-13.1)^b^	3.7 (1.8-7.4)	0.5	7.4 (3.4-15.1)^b^	2.8 (1.7-4.6)	0.7	4.0 (2.2-7.3)^b^
Heroin	0.8 (0.3-2.1)	0.2	4.0 (1.4-10.9)^b^	0.6 (0.2-1.3)	0.2	3.0 (1.2-8.2)^b^	0.5 (0.1-3.2)	0.2	2.5 (0.3-18.3)
Crystal meth^d^	1.0 (0.4-2.4)	0.3	3.3 (2.6-4.3)^b^	1.4 (0.8-2.7)	0.2	7.0 (3.1-14.9)^b^	3.3 (1.6-6.8)	0.4	8.3 (3.0-19.1)^b^
Cigarettes	10.6 (7.8-14.2)	2.6	4.1 (2.9-5.8)^b^	15.1 (10.4-21.3)	4.9	3.1 (2.1-4.7)^b^	23.1 (17.9-29.4)	10.5	2.2 (1.6-2.9)^b^

Abbreviations: LSD, lysergic acid diethylamide; MTF, Monitoring the Future; NA, not available; RR, relative risk.

^a^ Confidence intervals for MTF data can be found in Miech et al,^19^ Tables 4-1a through d.

^b^ *P* < .05.

^c^ Use of any illicit drug includes any use of LSD, other hallucinogens, crack, cocaine other than crack, heroin, any use of narcotics other than heroin (grade 12 only), amphetamines, or tranquilizers not under a physician’s order.

^d^ Numbers for MTF grades 8 and 10 are for methamphetamine (including crystal meth).

### Lifetime Prevalence

Lifetime rates of American Indian students for all substance measures except tranquilizers and amphetamines for each grade were higher than MTF rates at *P* < .05 (Table 1). The highest lifetime rates for American Indian students in grade 8 were for marijuana (43.7% [95% CI, 35.1%-52.7%]), alcohol (39.7% [95% CI, 31.4%-48.6%]), cigarettes (29.7% [95% CI, 22.4%-38.1%]), and having been drunk (22.9% [95% CI, 17.3%-29.7%]), with respective RRs of 3.4 (95% CI, 2.8-4.2), 1.7 (95% CI, 1.4-2.2), 3.0 (95% CI, 2.3-4.0), and 2.7 (95% CI, 2.0-3.5). Results for students in grades 10 and 12 were similar, but with greater reported use as grades increased for both American Indian and MTF students and lower RRs, although they were still significantly different than 1. For example, the American Indian students’ rate of marijuana use increased to 55.6% (95% CI, 46.7%-64.2%) by 10th grade, but its corresponding RR decreased to 1.9 (95% CI, 1.6-2.2).

Lifetime illicit drug rates excluding marijuana were 16.2% (95% CI, 12.7%-20.5%), 19.1% (95% CI, 12.8%-27.6%), and 24.4% (95% CI, 18.8%-31.0%) for American Indian students in grades 8, 10, and 12, respectively. The lifetime illicit drug RR for grade 8 was significantly different from 1 (1.8 [95% CI, 1.5-2.3]) but the RRs were not significantly different from 1 for grade 10 (1.4 [95% CI, 0.9-2.0]) or grade 12 (1.2 [95% CI, 0.9-1.5]).

### Last-30-Day Prevalence

As with lifetime use, for American Indian students in grade 8, last-30-day use rates for all substance measures except tranquilizers and amphetamines were higher than MTF rates at *P* < .05 (Table 2). The highest rates were for marijuana (22.5% [95% CI, 16.1%-30.5%]), alcohol (15.8% [95% CI, 10.7%-22.7%]), binge drinking (11.8% [95% CI, 6.4%-20.6%]), and cigarettes (10.6% [95% CI, 7.8%-14.2%]), with respective RRs of 4.2 (95% CI, 3.1-5.8), 2.1 (95% CI, 1.4-3.0), 3.5 (95% CI, 2.0-6.0), and 4.1 (95% CI, 2.9-5.8), respectively. These RRs (excluding binge drinking, which has no lifetime measure) are greater than those for lifetime use. As grade increased, last-30-day use for these measures increased for both American Indian and MTF students, but RRs decreased. The RRs for 10th and 12th graders for alcohol were not significantly different from 1, nor was the RR for 12th graders for having been drunk.

Last-30-day illicit drug use rates excluding marijuana for American Indian students in grades 8, 10, and 12 were 6.4% (95% CI, 4.6%-8.9%), 6.7% (95% CI, 3.8%-11.7%), and 9.7% (95% CI, 7.0%-13.3%), compared with respective MTF rates of 2.7%, 4.4%, and 6.9%. The RR associated with these rates for eighth graders was 2.4 (95% CI, 1.7-3.3), while the rates for 10th and 12th graders were not significantly different from 1 at *P* < .05. In addition, RRs for amphetamines and tranquilizers were not significantly different from 1, nor was the RR for LSD for 10th graders or the RR for heroin for 10th and 12th graders. All other RRs were significantly different from 1, with use by American Indian students being greater than use by MTF students.

### Change in RR From 2009-2012 and 2016-2017

The RR between American Indian and MTF students for lifetime alcohol use changed little from 2009-2012 (RR, 1.5 [95% CI, 1.4-1.6]) to 2016-2017 (RR, 1.3 [95% CI, 1.2-1.4]). This was also true for lifetime marijuana use (2009-2012: RR, 2.0 [95% CI, 1.8-2.1]; 2016-2017: RR, 2.1 [95% CI, 1.9-2.3]). However, lifetime RR for use of other drugs increased substantially across these years with an RR of 1.8 (95% CI, 1.7-1.9) for 2009 to 2012 and an RR of 3.0 (95% CI, 2.9-3.2) for 2016-2017. For last-30-day use, the RR increased for last-month alcohol use from 1.5 (95% CI, 1.3-1.7) to 2.1 (95% CI, 1.9-2.3), but no change was found for RR for marijuana use, with the RR at 2.8 for both points. The RR for other drug use increased from 2.5 (95% CI, 2.3-2.8) to 3.9 (95% CI, 3.6-4.2).

## Discussion

Findings from this study demonstrate that American Indian adolescents who reside on or near reservations continue a trend of using nearly all substances at substantially higher rates than adolescents from a nationally representative sample (MTF). Lifetime exposure was higher for American Indian reservation-based youths, with significant RRs compared with national youths. The only exceptions to this pattern were for amphetamines and tranquilizers at all grades and lifetime use of any illicit drug other than marijuana at grades 10 and 12. Similarly, higher rates for American Indian youths were found for current (last 30 days) substance use, with the exception of amphetamines and tranquilizers at all grades, alcohol at grades 10 and 12, having been drunk at grade 12, any illicit drug other than marijuana at grades 10 and 12, LSD at grade 10, and heroin at grade 12. The RRs decreased with grade in school; by grade 12, MTF students were more similar to American Indian students, but American Indian students’ rates remained an average of at least twice that of MTF students’ rates.

The higher rate of substance use among American Indian students compared with MTF students at grade 8 stresses the critical need for early prevention intervention efforts for American Indian youths living on or near reservations. Yet, few interventions have been developed and tested for this group.^21^ The distinct living environment of the reservation, coupled with high normative rates of use by peers and adults,^22^ creates prevention and treatment challenges unique to these youths. While American Indian youths are similar to other youths in many respects, with similar risk and protective factor profiles,^22,23,24^ these youths experience high rates of trauma and loss, such as suicide, accidents, violence, and substance abuse, in addition to other adverse childhood experiences such as child abuse and household dysfunction.^25,26,27^ Studies have established relationships between these experiences and higher rates of alcohol and drug use.^28,29,30,31^ Prevention efforts found to be effective in the general population may show less effectiveness in this population because of differences in childhood experiences and environment. Moreover, prevention efforts that do not attend carefully to cultural adaptation may not be acceptable, increasing the chance for failure.^32^ Cultural and value-based characteristics unique to American Indian populations, such as traditional Native American spirituality and the importance of expanded kinship networks, may provide beneficial targets for prevention programming, although there is still little etiologic evidence on how these work to prevent risky behaviors. These can be incorporated into existing evidence-based interventions or used in developing interventions from the ground up to create a portfolio of evidence-based, culturally grounded interventions.^32^ While the need for prevention and intervention is high and the research base is low compared with programs for other minority groups, scientific evidence is building. For example, under the Interventions for Health Promotion and Disease Prevention in Native American Populations (PAR-14-260), the National Institutes of Health have funded a number of studies to adapt, develop, and test substance use prevention programs with traditional Native American practices and cultural traditions to establish evidence-based practices for this population.^33^

Concurrent to increasing prevention efforts among this group, it is important to closely monitor and screen American Indian youths for substance use. High rates of both lifetime exposure and current use by eighth grade place these youths at enhanced risk for development of substance use disorders as well as other substance-related problems.^6,13,14^ Of particular concern are alcohol and marijuana, as 4 in 10 students have used alcohol, nearly 1 in 4 students have gotten drunk, and more than 4 in 10 have used marijuana. These rates are 1.7 to 3.4 times higher than for MTF students. Given new national legal statutes regarding recreational use of marijuana, changing attitudes of youths toward perceived harmfulness of marijuana may be associated with these alarming rates of use. To date, legalization appears to be increasing marijuana use among young people already using, not among nonusers.^34^ There is also evidence that legalization is increasing use among heavy alcohol users.^35^ While the legal status of cannabis, both at the federal and state levels, remains in flux, some reservations are considering moves toward legalization.^36^

For American Indian youths, early initiation and the combined use of alcohol and marijuana were associated with increased risk for abuse or dependence from 2 to 5 times compared with those who use only 1 of these substances.^9^ Physicians and other medical staff treating American Indian youths (eg, Indian Health Service) need to be particularly alert to screen for emerging and established substance use, abuse, and dependence among both younger and older American Indian adolescents. In light of the general public’s lowering of perceived harm of marijuana use, a helpful resource for medical and other treating staff is the recently published volume from the National Academy of Sciences regarding health effects of cannabis and cannabinoids.^37^ This volume provides a comprehensive and fair presentation of both therapeutic and negative health effects of cannabis, along with the most currently available medical evidence.

The other disturbing trend from these data is that the gap between American Indian and MTF adolescents in other illicit drug use appears to have increased substantially from 2009-2012 to 2016-2017.^6^ Comparative rates for cocaine, crystal meth, and psychedelics other than LSD are particularly troubling, ranging from 7.3 to 8.3 times higher than the current national MTF sample. While absolute rates for last-30-day use remain low for substances other than alcohol and marijuana (none >10% for all other drugs), much higher rates for American Indian youths signal increased risk for this group and, along with alcohol and marijuana, warrant careful screening. While the current data are from reservation-based American Indian youths, the rate of mobility between reservations and urban and suburban areas is high among American Indian individuals.^38^ Thus, even though medical facilities may not be on or adjacent to a reservation, medical encounter with American Indian populations remains possible, and frontline and treating staff in these nonreservation locations should remain attentive to the specific substance use screening needs of American Indian youths.

### Limitations

Our study had limitations. Although we used a stratified random sample of schools located on or near American Indian reservations, school participation in the survey was voluntary. If drug use in participating schools was significantly different than drug use in nonparticipating schools, nonresponse bias in the drug use estimates will be present. However, the decision to participate in the OYOF survey often depends on circumstances specific to events at the school unrelated to drug use, such as another study being required by the district that year or reduced instructional time due to a weather event. Participating schools were reasonably representative of sampled schools in several measurable characteristics. In addition, as with MTF students, most variation in dependent variables such as drug use is within schools, not between schools.^39^ This is the only national sample of American Indian youths living on or near reservations of which we are aware, and every effort was made to ensure a representative sample of schools.

Student participation in the survey at each school was not 100%, due to opt outs (<1%), absenteeism, and other factors. However, 87% of enrolled students completed the survey, a rate similar to MTF students. Students with high absenteeism are more likely to be drug users compared with those with low absenteeism.^40,41^ Thus, some degree of bias is present in the prevalence rates without these absentees. Estimates from MTF also reflect this bias; given similar survey participation rates by students and if absentees occur for similar reasons across the MTF and American Indian samples, the relative risks will be relatively free of this bias. Also not included in the sample are youths who have dropped out of school. Given the high rate of school dropout among American Indian youths, our results likely underestimated the substance use rates for adolescents in grades 10 and 12.^42^

## Conclusions

Our results clearly indicate that American Indian youths residing near or on reservations are at high risk for substance use, with the gap between these American Indian youths and US youths in general showing no sign of narrowing. By continuing to monitor and report rates of substance use among this population, we hope not only to continue to inform key audiences about this issue, but also to spur action and increase resources for prevention and intervention. The costs to these youths, their families, and their communities is simply too high for these disparities to continue.^32^
